# Strategic Adhesion and Dental Tissue Conservation: Contemporary Perspectives on Interfacial Bond Longevity and Minimally Invasive Restorative Designs

**DOI:** 10.3390/biomedicines14061391

**Published:** 2026-06-19

**Authors:** Cristiana Cuzic, Mihai Rominu, Horatiu Urechescu, Alisia Pricop, Ovidiu Stefan Cuzic, Raul Rotar, Marius Octavian Pricop, Anca Jivanescu

**Affiliations:** 1 Department of Prosthodontics, Faculty of Dental Medicine, University of Medicine and Pharmacy “Victor Babeș”, 300041 Timișoara, Romania; pricop.cristiana@umft.ro (C.C.); rotar.raul@umft.ro (R.R.); jivanescu.anca@umft.ro (A.J.); 2 TADERP Research Center—Advanced and Digital Techniques for Endodontic, Restorative and Prosthetic Treatment, University of Medicine and Pharmacy “Victor Babeș”, Revolutiei Ave. 1989, No. 9, 300041 Timișoara, Romania; 3 Research Center in Dental Medicine Using Conventional and Alternative Technologies, School of Dental Medicine, University of Medicine and Pharmacy “Victor Babeș”, 300070 Timișoara, Romania; rominu.mihai@umft.ro; 4 Department of Prosthesis Technology and Dental Materials, Faculty of Dentistry, University of Medicine and Pharmacy “Victor Babeș”, 300041 Timișoara, Romania; 5 Department of Oral and Maxillo-Facial Surgery, Faculty of Dental Medicine, University of Medicine and Pharmacy “Victor Babeș”, 300041 Timișoara, Romania; pricop.marius@umft.ro; 6 Department of Overland Communication Ways, Foundations and Cadastral Survey, Politehnica University Timisoara, No. 2 Traian Lalescu Str., 300223 Timisoara, Romania; ovidiu.cuzic@student.upt.ro

**Keywords:** minimally invasive prosthodontics, all-ceramic restorations, adhesion, resin cements, cementation protocol, computer-aided design, modern dentistry

## Abstract

Modern prosthetic dentistry has been significantly reshaped by adhesive dentistry, CAD/CAM technologies, and advanced ceramic materials, leading to the development of minimally invasive all-ceramic restorative approaches. However, the longevity of the adhesive interface is fundamental to the long-term effectiveness of these restorations. With a focus on bond durability and clinical performance, this narrative review aims to evaluate modern adhesive strategies, tooth preparation requirements, and cementation techniques in all-ceramic minimally invasive restorations. **Methods**: A narrative review of the literature was performed using Google Scholar, Web of Science, and PubMed/MEDLINE databases. Publications from 2000 to 2026 were analysed. In vitro research, narrative reviews, and systematic reviews related to adhesive systems, resin cements, CAD/CAM materials, and minimally invasive prosthodontic principles were the core subjects of the research. **Results**: The findings indicate that material selection, surface conditioning techniques, and cementation methods have a significant impact on the clinical effectiveness of all-ceramic restorations. Retention and marginal sealing are greatly enhanced by resin-based adhesive systems. Nevertheless, hydrolytic degradation, procedure sensitivity, and substrate-related factors remain a challenge to the adhesive interface. Advances in CAD/CAM and ultra-conservative designs, like occlusal veneers and partial-coverage restorations, have increased treatment alternatives while ensuring acceptable functional and aesthetic results. **Conclusions**: Minimally invasive all-ceramic restorations represent a conservative and clinically effective treatment approach in modern prosthodontics. Their long-term performance is primarily dependent on adhesive interface stability and adherence to evidence-based clinical protocols. Continued developments in adhesive materials and ceramic systems are expected to improve bond durability and broaden clinical indications.

## 1. Introduction

Finding the perfect balance between aesthetics and function is often the primary objective in restorative and cosmetic dentistry, especially for patients looking for procedures to address aesthetically related problems. The overall appearance of the face and smile is greatly influenced by the anterior area of the maxilla. It is becoming more and more difficult for dentists to provide treatments that satisfy patients’ high cosmetic standards while also addressing challenges with functionality. The development of sophisticated restorative materials and procedures has given clinicians the ability to produce outcomes that are both highly attractive and long-lasting, in response to the growing desire for less invasive therapies [1].

The aesthetics and biomechanics of minimally invasive prosthodontic restorations are factors that contribute to patient satisfaction and the long-term effectiveness of the restorations [2]. Anterior restorations are commonly designed using mathematical criteria like the Golden Proportion and Berry’s Biometric Index [3]. These indicators guarantee that anterior tooth width, height, and spacing are aesthetically correct and effective. Translucency, a fundamental factor in aesthetic restorations, is determined by factors like enamel thickness and light transmission [4].

Adhesive approaches have transformed restorative and prosthetic dentistry. The implementation of conservative treatment methods reduces tissue damage and improves pulp vitality [5]. Research on adhesion processes and novel biomaterials has broadened the use of bonded partial restorations. Bonded restorations are a solid alternative to full-coverage crowns, even in severe decay instances, without requiring tissue mutilation or periodontal aggressiveness [6].

Bonded restorations may improve coronal retention without reducing root anchoring, making pre-prosthetic endodontic treatments unnecessary [7], providing substantial benefits in sustaining pulp vitality and exceeding conservative treatment limitations [8].

The investigation of residual structure will be the primary element in selecting a therapy. Occlusal and aesthetic context, as well as tooth placement, are other important aspects to consider [9]. Molars have a larger bonding surface and pulp chamber, and mastication is mostly compression-based, making them more advantageous than premolars. In contrast, premolars experience more damaging lateral shearing stresses [10]. Anterior teeth present unique adhesive and biomechanical challenges that differ substantially from those of posterior teeth. Although the available bonding surface is generally smaller than in molars, anterior restorations often benefit from a high proportion of enamel, which provides a more predictable and durable bonding substrate. In addition, treatment planning in the anterior region must balance biomechanical requirements with aesthetic integration, phonetics, and preservation of dental tissues. The selection of restorative designs, therefore, extends beyond mechanical considerations alone and should be guided by a strategic assessment of functional risk, remaining enamel, and aesthetic objectives. Consequently, minimally invasive adhesive restorations have become particularly valuable in anterior teeth, where tissue preservation and long-term bond durability are closely interconnected [11].

In many clinical contexts, minimally invasive restorations are not suitable. The clinician must carefully assess each case to determine if the specific indications and circumstances demand such types of restorations, in which case they may need additional processes in treatment planning and implementation compared to conventional restorations, as well as heightened skills and precision during fabrication and fitting, along with specialised maintenance and repair methods [12]. 

Adhesive failure, as it relates to minimally invasive all-ceramic restorations, is the deterioration or loss of the bond between the restoration and the tooth structure [13]. This may occur at several interfaces, including the tooth–restoration interface and the adhesive–resin junction [14]. Adhesive failure can affect the durability and stability of restorations, possibly resulting in both aesthetic and functional issues [15].

Patients should be educated on proper dental care to ensure the longevity and efficacy of adhesively retained prosthodontic restorations. Follow-up visits and the clinician’s maintenance define the monitoring of the status of the restorations and addressing any issues as soon as possible. When conventional mechanical retention is limited or impossible, adhesive-bonded prosthodontic restorations are a useful therapeutic choice. These restorations can offer stability, functionality, and aesthetics while preserving tooth structure. The clinician’s ability and the patient’s cooperation are aspects of assuring the clinical durability and efficacy of minimally invasive prosthodontic restorations [15].

**Figure 1:**
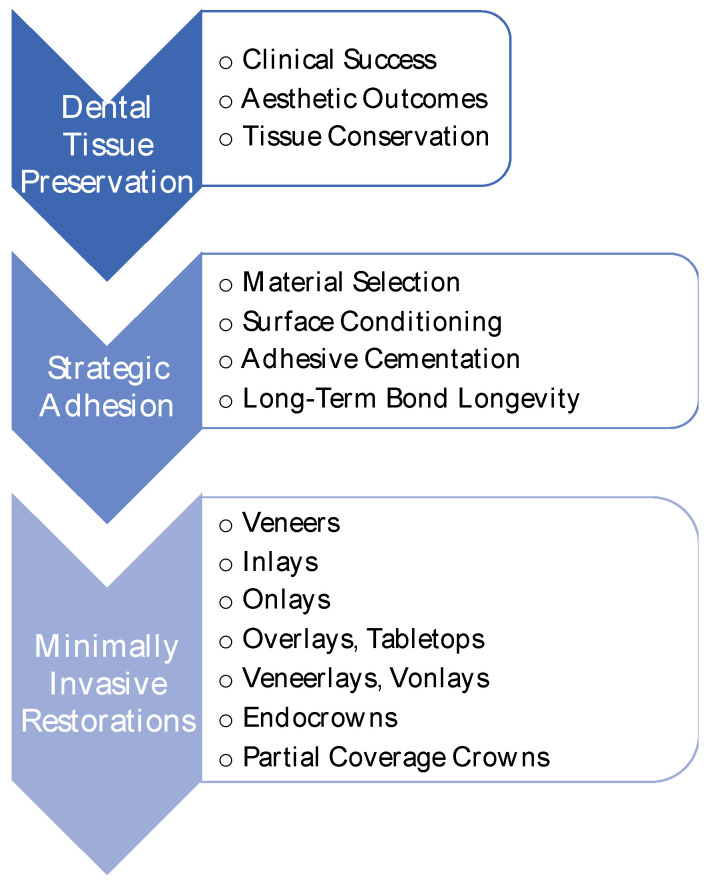
Conceptual framework of strategic adhesion and dental tissue conservation in minimally invasive ceramic restorations.

Adequate case examinations, treatment planning, appropriate patient education, preventative strategies, and the dentist’s recommendations for the patient, along with frequent follow-up appointments and restoration monitoring—these are all measures taken by practitioners to enhance the lifetime of restorations [16]. The aim of this narrative review is to provide a comprehensive overview and to critically synthesise current evidence of the advanced adhesive bonding procedures, precise tooth preparation strategies, and defined cementation protocols in all-ceramic minimally invasive restorations (Figure 1), with a focus on interfacial bond stability and long-term clinical performance.

The objective is to evaluate contemporary advances in adhesive dentistry and restorative materials, emphasising their impact on treatment predictability, structural preservation, and aesthetic outcomes in modern prosthodontic practice. Understanding these qualities and their impact on restoration success or failure is essential for dental practitioners in achieving effective and enduring treatment outcomes.

## 2. Materials and Methods

This narrative review explores current evidence on adhesive strategies and restorative designs in all-ceramic prosthodontics within the context of minimally invasive dentistry. The literature was identified through a narrative search of PubMed/Medline, Web of Science, and Google Scholar databases, covering publications from 2000 to 2026.

The search strategy included combinations of the following keywords and their variations: “all-ceramic restorations”, “adhesion”, “cementation protocols”, “resin cements”, “CAD/CAM dentistry”, and “minimally invasive prosthodontics”.

Eligible studies included in vitro investigations, narrative and systematic reviews, and comprehensive clinical evaluations addressing adhesive mechanisms, ceramic materials, and restorative designs in digital dentistry. Narrative and systematic reviews were included to ensure contextual coverage of the topic and to synthesise evidence from areas where primary studies are heterogeneous or scarce. In vivo studies reporting solely on indirect clinical outcomes without assessment of adhesive mechanisms were not prioritised; however, comprehensive clinical evaluations providing systematic outcome data across patient populations were included to complement experimental in vitro evidence. Case reports were excluded due to their isolated nature and low level of evidence. Non-English publications were also excluded.

From the initial search results, 61 studies were selected for detailed qualitative analysis based on relevance to the topic and scientific contribution. An additional set of supporting references (*n* = 59) was used to strengthen contextual interpretation and ensure comprehensive coverage of the topic, resulting in a total of 120 references included in the review.

The synthesised evidence was organised thematically according to (i) adhesion strategies for all-ceramic restorations and (ii) minimally invasive prosthetic designs and restorative applications.

## 3. Adhesion Strategies for All-Ceramic Restorations

The use of highly advanced adhesive dentistry before the development of CAD/CAM technology in dentistry deserves to be recognised. The clinical implementation of CAD/CAM-generated prostheses would not have been possible without adhesive technology. Additionally, the CAD/CAM treatment and an intraoral scanner allow for the same-day execution of the semi-direct treatment, which is unquestionably an indirect technique. Consequently, the distinctions between direct and indirect approaches are diminished. This development has contributed to the concept of “full digital treatment”. Currently, preparation and luting continue to be analogue operations. Adhesive dentistry significantly influences traditional methods and is essential for the development of a truly entirely digital prosthodontic approach [17]. There are a considerable number of CAD/CAM blocks available, and the goal of locating them is to make building all kinds of restorations easier [18]. Selecting materials for dental applications depends on factors such as strength, aesthetics, precision, and strong bonding to substrates.

### 3.1. Resin-Based Luting Systems and Cementation Principles

**Figure 2:**
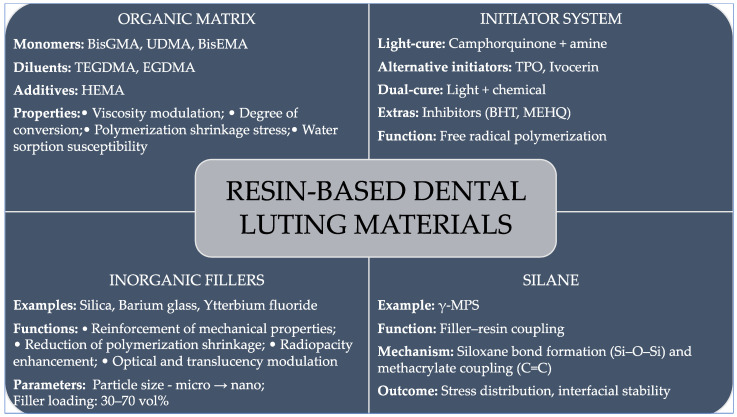
Chemical Composition of Resin-Based Luting Systems.

Demand for ceramic indirect restorations in everyday dentistry has developed significantly in recent years due to patients’ increasing importance for aesthetics and some patients’ reactions to dental metals [19]. The adhesive resin-based cement’s mutually beneficial bond with the tooth’s support structure and ceramic restoration strengthens and improves the entire system, which leads to a successful prosthodontic restoration [20]. Even so, there is still an active strategy movement on the best ceramic restoration cementation protocol. Results in general dental practice suggest that conventional and resin-based cements can cement polycrystalline ceramic restorations. Some practitioners choose conventional cements since they do not require dental tissue pretreatment or handling protocols and they tolerate moisture well. In the course of time, though, the bond between the restoration and the tooth structure weakens. The chemical composition of resin-based luting systems is listed and summarised in Figure 2. 

However, resin cements have superior aesthetic and mechanical properties and provide an effective and long-lasting adhesive bond, making them a trustworthy solution for polycrystalline ceramic restorations [19,20,21].

### 3.2. Adhesion Mechanisms and Cementation Protocols for CAD/CAM Ceramics

Clinical practitioners used zinc-phosphate cement from the conventional cement group for cementing gold and non-precious metal restorations for generations. Clinical investigations have shown restoration long-term performance of up to twenty years, justifying its usage for cementing crowns and bridges [22,23]. After polycarboxylate and glass ionomer cements were created in the early 1970s, Hecht and Ludstech developed resin cements for ceramic restorations in 2002. Their good mechanical performance, solid adhesive bond with tooth structure and restoration, easy handling, and satisfactory aesthetics have been reported [24,25]. Due to their superior tooth adherence, adhesive cements are superior luting materials. The quality of cementation, the final step in performing indirect restorations (metal–ceramic crowns and bridges, full ceramic crowns and bridges, inlays, onlays, and fibre posts) majorly impacts success in clinical practice [26].

Cementing techniques may be classified as adhesive or non-adhesive [27]. Adhesive cementation combines chemical bonding and micromechanical interlocking, requiring a special agent to help adhere the restorative material to the surface [28]. Three varieties of resin cement can be distinguished by their chemical interactions with tooth tissue: non-adhesive, chemically bonded, and micromechanically bonded cement [29]. Non-adhesive or traditional cementation relies only on micromechanical retention and involves the use of a luting agent to seal the area between the restoration and the natural tooth. The ceramic composition, preparation retention and resistance shape, and field management during cementation all affect the prescriptions for certain cementation procedures [30]. Conventional cementation is still a widely used method for dental restorations, but with limitations. Surface treatment is not necessary for non-adhesive cement on a tooth or restoration, meaning it is used for thicker ceramic restorations (1.5 mm to 2 mm) or porcelain-fused-to-metal prosthodontic restorations [31].

In complex restorations, a luting agent for micromechanical retention may be insufficient. Adhesive cementation is advantageous in this context, relying on both micromechanical and chemical adherence between the restoration and the dental structure. This enhances retention and resistance to debonding, making it suitable for challenging situations. However, careful preparation of the tooth surface and the use of certain bonding agents are necessary. The choice between conventional and adhesive cementation is influenced by factors like the kind of ceramic, the retention and resistance form of the preparation, and the management of the field throughout the cementation process. Dentists may guarantee successful outcomes for their patients’ restorations by understanding these factors and choosing the appropriate method [32,33].

Adhesive cementation will improve short, tapered preparations by creating a dentin hybrid layer that optimises the mechanical retention of the restoration. Nonetheless, the use of bonding agents necessitates additional procedures and rigorous isolation, which may be problematic in a clinical setting. Furthermore, practitioners must ensure that the laboratory team follows precise methods to achieve proper adaptation since adhesive cement cannot rectify inadequate fit [32,34].

Adhesive systems have been divided into several standardised classifications. The number of clinical steps and the mechanisms of the procedure itself have changed over the generations of luting dental materials [35].

#### 3.2.1. Multi-Step Adhesive Systems

- Etch-and-Rinse (Total-Etch) Systems.

Typical etching and rinsing adhesive cements follow the same clinical stages as the total-etch adhesive system. According to Vincent et al. [5], there are a minimum of three distinct phases in this adhesion technique. Primarily, selecting a conditioner or acid etchant; subsequently, applying a primer or adhesion-activating agent; and last, adding the bonding agent or adhesive resin. The second and third processes are combined in a reduced form, although there is still a distinct “etch and rinse” phase [7], so this method is dependent on only two steps. First, in order to condition the surface and create additional roughness, hydroxyapatite crystals are selectively dissolved by etching, often by using a 30–40% phosphoric acid gel. In the second phase, individually exposed hydroxyapatite crystals are covered by in situ polymerisation of resin that is easily absorbed by capillary attraction within the etch peaks that have been created [36]. This allows cement to penetrate further into the porosities [27].

The primary bonding mechanism of etch-and-rinse adhesives to dentin depends on the resin’s infiltration and adhesion to the collagen fibre structure, a process that has to be as comprehensive as possible [35].

b. Self-Etch Systems.

The self-etch technique is considered a favourable alternative simply due to its ease of execution and lower susceptibility to technique errors. In contrast to conventional techniques, it avoids the demand for a distinct etching step, so no rinsing is necessary in this instance, and an acidic monomer will diffuse through the smear layer [37], therefore optimising the workflow and minimising the incidence of making mistakes. The long-term implications of integrating dissolved hydroxyapatite crystals and residual smear layer areas inside the bond have still not been sufficiently addressed. An excessive amount of primer/adhesive agent present in the interfacial matrix may inhibit monomer polymerisation or contribute to nanoleakage, thereby weakening the bond integrity, meaning the hydrophilic interfacial structure is vulnerable to hydrolytic degradation.

According to Van Meerbeek et al. [38], self-etch adhesives feature three different acidic pH levels: ultra-mild (pH > 2.5), strong (pH 1.0), and mild (pH 2.0). This approach can be one-step or two-step: a strong pH initially focuses on diffusion-based bonding mechanisms, while mild self-etch adhesives offer the best bonding performance to dentin by creating a demineralisation layer of the surface through hybridization [5]. Dentin is dynamic and difficult to bond with due to its characteristics and factors that impact the dentin’s permeability. Vasoconstrictors reduce pulpal pressure and fluid flow in the tubules, but various other variables like tubule size and length, dentin fluid viscosity, pressure variation, volume of dispersed elements in tubular fluid, and pulp blood vessel removal rate influence permeability [39].

Mild self-etch adhesives have potential; nevertheless, their primary limitation is in their bonding capacity to enamel, whereas robust self-etch adhesives provide a balance between demineralisation depth and bonding efficacy [40]. Investigations and studies are still assessing the long-term durability and efficacy of self-etch techniques.

c. Universal adhesive systems.

The development of multi-mode (universal) adhesives in modern dentistry aims to simplify clinical protocols by integrating the principles of both etch-and-rinse and self-etch strategies within a single system. Unlike earlier one-step self-etch adhesives, universal adhesives are designed for a wider range of clinical applications, including direct and indirect restorations, bonding to zirconia, and resin coating procedures [7,41].

A key determinant of their performance is the incorporation of the functional monomer 10-methacryloyloxydecyl dihydrogen phosphate (10-MDP). This monomer enables mild self-etching of dental substrates while establishing a chemical interaction with residual hydroxyapatite [42]. However, its etching efficacy on enamel is limited compared to phosphoric acid, which justifies the frequent recommendation for selective enamel etching in clinical practice [43].

The functional advantage of 10-MDP is largely attributed to its relatively low hydrophilicity and its ability to form stable calcium salts (MDP-Ca). These nanolayered structures are considered to contribute to the durability of the adhesive interface by reducing hydrolytic degradation and reinforcing the hybrid layer [44]. Nevertheless, the extent to which these chemical interactions translate into long-term clinical stability remains influenced by multiple factors, including substrate characteristics and application protocol.

Despite their versatility, universal adhesives remain susceptible to degradation in the oral environment. Long-term studies have shown that, although the self-etch mode may reduce nanoleakage compared to more aggressive strategies, bond durability can still decline over time, particularly under prolonged mouth conditions. Similar degradation patterns have been reported for earlier self-etch systems [45]. Clinical evidence suggests that, for certain universal adhesives, the self-etch approach may be associated with increased marginal staining compared to etch-and-rinse or selective enamel etching protocols, although outcomes are material- and technique-dependent [46].

Recent formulations, such as 3M™ Scotchbond™ Universal Plus Adhesive (3M Oral Care, St Paul, MN, USA), incorporate advanced silane chemistry aimed at improving adhesion to silica-based ceramics. The inclusion of both 3-(aminopropyl) triethoxysilane (APTES) and γ-methacryloxypropyltriethoxysilane (γ-MPTES) is intended to enhance silanisation efficiency and stability, potentially overcoming limitations associated with the hydrolytic instability of conventional single-silane systems [47]. However, the clinical significance of these modifications continues to be evaluated.

#### 3.2.2. Self-Adhesive Resin Cements

The development of self-adhesive resin cement offers an alternative for multiple-step resin cementation protocols. By eliminating the need for separate etching and bonding procedures, surface conditioning is not required in principle. However, additional pretreatment strategies may still be employed to enhance bonding effectiveness in specific clinical situations. In order to improve a self-adhesive reaction, the adhesive interaction between this type of cement and the tooth structure, self-adhesive resin cements contain additional multifunctional methacrylate monomers with phosphoric acid groups, which enable simultaneous demineralisation and infiltration of the dental substrate. Due to its implementation, a low pH value and increased hydrophilic characteristics can be achieved from the initial stage of the setting process, facilitating interaction with enamel and dentin. The negatively charged monomer groups interact chemically with the tooth’s calcium ions from residual hydroxyapatite, which, when combined with the filler’s alkaline component, induces a neutralisation process [48]. This objective is a consequence of thorough investigations in dental luting materials, leading to upgraded luting agents, particularly through the advancement of contemporary resin adhesive cement [49]. Additionally, phosphoric acid methacrylate (with a pH below 2.0) can partially demineralise dental minerals and substitute them with cement. However, unlike conventional adhesive systems, self-adhesive resin cements generally do not form a well-defined hybrid layer, leading instead to a more superficial and heterogeneous interaction zone [31,50,51]. Al-Assaf et al. [52] consider the accumulation of the smear layer in the interface affects the bonding performance and will result in a lower bonding strength compared to various types of adhesive cements that involve prior substrate conditioning.

Eugenol-containing temporary cements prior to adhesive procedures are not recommended and generally discouraged due to a potential decrease in bonding, as eugenol has been shown to interfere with free-radical polymerisation, particularly when dentin is contaminated [53]. Proper and effective cleaning and decontamination of the preparation will define successful bonding, which can be achieved through low-pressure and small-particle air abrasion followed by water spray [53,54]. These procedures aim to remove residual substances and contaminants, thereby improving substrate reactivity and future adhesive performance.

Colour differences between try-in pastes and their corresponding cured resin cements can be attributed to the distinct compositions and optical properties. In clinical practice, try-in pastes are used as a guide to select the most appropriate resin cement shade, with the goal of achieving a visually pleasing and aesthetically harmonious integration between the final porcelain veneer restoration and the adjacent natural dentition. Still, given that measurable colour differences exist between try-in pastes and their corresponding cured resin cements, the final restoration may appear clinically unacceptable if careful shade verification at the time of definitive cementation is not performed. Therefore, try-in pastes should be regarded as a preliminary approximation rather than a definitive predictor of the final aesthetic outcome [55].

#### 3.2.3. Adhesive Cementation Strategies

Glass ceramics, low-filled glass ceramics, and intermediate-filled glass ceramics all have the potential to develop an adhesive bond by applying resin luting agents. Current research additionally indicates that the use of dental luting materials based on composite resins contributes to the ceramic material’s fracture strength because of the beneficial effects of their mechanical qualities (hardness, flexural strength) on the ceramic restoration’s resistance to fracturing and the strength of the restored tooth [56,57,58]. Different ceramic substrates require distinct pretreatment strategies in order to optimise micromechanical retention and chemical bonding with resin-based luting systems. The main surface conditioning methods and adhesion mechanisms associated with contemporary CAD/CAM all-ceramic restorative materials are summarised in Table 1.

- Pretreatment technique for predominantly glass ceramics:

Conventional cementation is inadvisable for feldspathic ceramic [59]. The clinician must prepare glass feldspathic ceramics for adhesive cementation by etching the intaglio surface with a hydrofluoric (HF) acid solution at concentrations between 5 and 10%. This method enhances surface area, optimises micromechanical retention, and ensures a clean surface for adhesive cementation. The use of silane on the etched surface boosts the wettability of resin cement and chemically interacts with both the resin matrix and the hydroxylated porcelain surface [32,60]. Both etching and silanisation are recommended [61].

Two distinct types of silanes are available: hydrolysed silanes, which are often one-bottle systems, and unhydrolyzed silanes, which are two-bottle or mixable systems that promise a fresh and active silane [62]. Adhesive cementation to enamel or dentin necessitates the use of an adhesive system, followed by the application of resin cement [28]. Self-etching adhesive solutions are readily accessible and user-friendly; nonetheless, they exhibit inferior bond strength to enamel compared to total-etch methods [63]. The total-etch three-step adhesive approach is considered the primary option for adhesive pretreatment [32].

- Pretreatment technique for particle-filled glass ceramics:

Low-filled materials containing leucite (IPS Empress Esthetic, Ivoclar Vivadent, Schaan, Liechtenstein or OPC, Jeneric/Pentron Inc., Wallingford, CT, USA) need adhesive bonding for their strength. For the type of ceramic that is mostly made of glass, the cementation method is the same as that which has already been discussed above [64]. Intermediate-filled materials reinforced with lithium disilicate (IPS e.max Press Ivoclar Vivadent, Schaan, Liechtenstein or OPC 3G Jeneric/Pentron Inc., Wallingford, CT, USA) offer strength and durability. This material may be cemented either adhesively or traditionally when used as full-coverage crown restorations. Traditional cementation is carried out without intermediate agents, using standard luting agents such as resin-modified glass ionomer cement. Adhesive cementation is required for partial-coverage or minimally invasive restorations for better retention as well as fracture resistance. Concise, clinically insufficient compositions should be adhesively bonded. Practitioners must provide appropriate isolation to maintain a contamination-free work area when using adhesive cement. To achieve optimal adhesion, the clinician must modify the conditioning technique of the restoration’s intaglio surface during the adhesive cementation of particle-packed glass ceramics. Manufacturers recommend etching the intaglio surface of leucite-reinforced restorations for about 60 s before cementation with a 10% hydrofluoric acid solution. Etch a lithium disilicate-reinforced ceramic for about 20 s using a 5% HF acid solution, followed by applying silane and resin cement, using a procedure similar to that used for most glass ceramics. Another type of particle-filled glass ceramic is composed of molten glass inserted in a sintered aluminium oxide core. As etching with HF acid seems to have barely any impact on resin cement retention, these restorations are bonded using traditional methods instead of adhesive techniques. An alternative cementation approach involves treating the ceramic with tribochemical silica and air abrading the intaglio surface, followed by the application of 10-MDP prior to resin cement application, thereby optimising its adhesion to this ceramic type [65,66,67,68,69].

- Pretreatment technique for polycrystalline ceramics:

Polycrystalline ceramics have specific characteristics since they do not include glass. Among them, zirconia-based ceramics have gained particular relevance in contemporary restorative dentistry due to their favourable mechanical properties, biocompatibility, and the development of highly translucent formulations suitable for aesthetically demanding clinical situations. Although polycrystalline ceramics are traditionally cemented using conventional approaches, adhesive cementation may provide additional benefits under specific clinical conditions.

In order to strengthen the bond of adhesive or self-adhesive resin cement, air abrasion with aluminium oxide or tribochemical silica followed by an adhesion-promoting agent (silane or ceramic primer) has been recommended [70]. Air abrasion optimises the accessible surface area for adhesion while also creating microcracks or potential fracture initiation points. Consequently, while low-pressure abrasion is recommended, the use of post-sintering surface treatments remains controversial in comparison to traditional cementation [71]. These adhesion-enhancing chemicals are thought to establish chemical bonds with zirconium oxide [71]. For improved retention, it has been considered to use air abrasion with 50-micrometre aluminium oxide powder at 7.0 pounds per square inch, followed by the application of a primer containing MDP prior to the application of resin cement [32]. In the absence of acidic adhesive monomers like 10MDP, silanes are unable to establish a chemical bond with zirconia [2].

**Figure 3:**
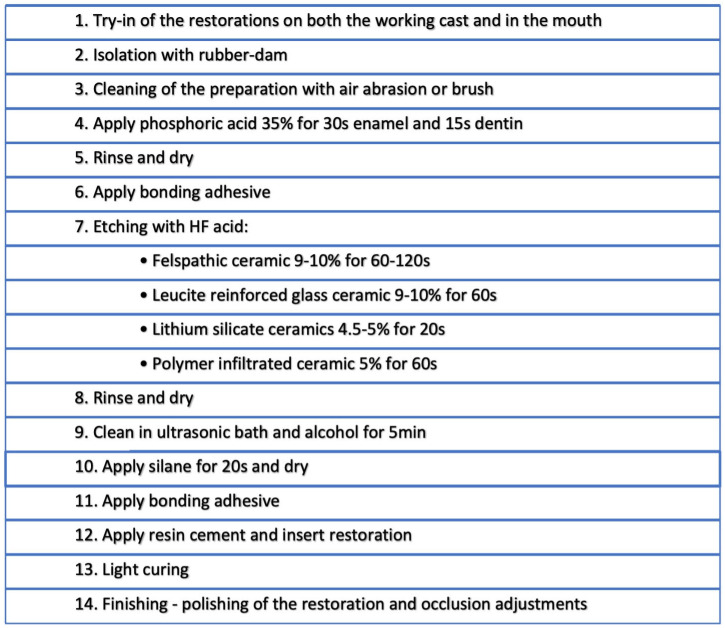
Clinical Sequence for Adhesive Bonding in Ceramic Adhesive Dentistry.

Surface treatments impact the clinical outcomes of all-ceramic restorative materials. Hydrofluoric acid etching combined with silanisation offers superior bond strength compared to other etchants. Etching and priming are recommended for strengthening bond strength in traditional adhesives and self-adhesive cements. Clinicians must select appropriate materials and cementation procedures for optimal aesthetic, functional, and long-term outcomes [72]. Clinical steps are detailed in Figure 3.

Clinical conditions need specific adhesion techniques in daily practice; under ideal circumstances, preparation margins are situated supragingival. The selection of cement is also affected by the preferences of the clinician and the restorative material used. For total-etch resin cements to be used successfully, the dental team needs to be consistent with the isolation capacity of the working area. The best seal and durability of restorations may be achieved using resin cements and total-etch procedures, but therapeutic effectiveness depends on rigorous clinical steps.

## 4. Minimally Invasive Prosthetic Strategies

Tooth preparation techniques for minimally invasive all-ceramic restorations offer numerous advantages for the preservation of natural tooth structure, aesthetic and functional improvements, and long-term durability. In restorative dentistry the concept has advanced from conservative to ultra-conservative [73], resulting in requiring the use of adhesive techniques that boost the bonding between ceramic and resin materials and tooth structure.

In order to achieve a high bonding strength and shorten the clinical steps, numerous companies continue to provide a variety of systems today. Still, selecting one of them still demands an extensive amount of experience and can get confusing.

Nevertheless, contraindications along with unique characteristics of patients must be carefully evaluated to provide optimal therapy outcomes. Understanding the various tooth preparation techniques and their indications is key for achieving excellent outcomes with minimally invasive restorations for all-ceramic dental restorations [74]. 

### 4.1. Adhesively Retained Partial-Coverage Restorations

The main objective of restorative dentistry is to preserve and sustain the natural tooth structure. Clinicians, together with patients, have selected partial-coverage ceramic restorations to meet their increased aesthetic expectations [75]. Various factors, including caries, erosion, abfraction, attrition, and fracture, can cause enamel loss, requiring further prosthodontic treatments [76].

Undiagnosed dental erosion is common due to gradual, progressive, and painless mineral loss, generally identified after significant tissue loss. To maintain the balance between biological, functional, mechanical, and aesthetic considerations, conservative treatment is recommended to preserve stable tooth structure [77].

Adhesive indirect prosthodontic restorations use alternative, unconventional retention strategies, such as undercuts or retention grooves, instead of standard mechanical retention methods [10]. These restorations aim to offer stability and support without requiring mechanical interlocking. Adhesively bonded prosthodontic restorations are often recommended when conventional mechanical retention is limited or insufficient, such as with

- Abutment teeth exhibiting limited tooth structure as a result of excessive decay, previous restorations, or dental trauma;
- Teeth with short clinical crowns, as a consequence of tooth wear, erosion, or attrition [12].

The clinical approach includes comprehensive treatment planning and the selection of appropriate techniques and materials. A detailed assessment and evaluation of the patient’s dental condition, such as the analysis of remaining tooth structure, periodontal health, occlusion, and aesthetic requirements, are needed [9]. The treatment plan is established based on the examination findings, taking into consideration the patient’s specific needs and the clinical context. Precise planning and adjusting of these restorations are required to produce harmonic occlusal contacts and force distribution during biting and chewing. Revisions and appropriate occlusal evaluation may be recommended to maximise the functional aspects of the restorations [12].

There are various restoration types, including inlays for cavities without cuspal coverage, onlays for cavities requiring cusp coverage, overlays for complete cuspal coverage, veneerlays for buccal wall involvement, endocrowns for pulp chamber retention, and full-coverage crowns [78].

Technological developments in CAD/CAM materials resulted in the application of occlusal veneers as a conservative alternative to conventional overlays or full-coverage crowns for restoring missing tooth structure on the occlusal surface [79]. Based on the characteristic strength and wear capabilities of materials like high-performance composite resins and lithium disilicate, thinner designs—also known as ultra-thin occlusal veneers—are now possible [80]. Research indicates that patients increasingly favour treatment approaches involving minimal or no additional tooth reduction after a treatment planning consult [81].

It is essential to understand the different tooth preparation processes and their indications in order to obtain effective results with minimally invasive restorations for all-ceramic prosthetic restorations.

- The inlay technique involves removing decayed or damaged tooth structure and putting a customised restoration into the obtained tooth cavity. This approach is particularly indicated for moderate-to-severe dental decay, cracked or weakened tooth structure, and replacing large amalgam or composite fillings to restore function and aesthetics while preserving healthy tooth structure. Advantages of inlays include preserving tooth structure, precise and custom-fit restorations, and excellent aesthetics.
  Inlays are contraindicated for severe dental decay, structural damage requiring full-coverage restorations, and inadequate tooth structure for retention and stability.
  An inlay is an extraoral dental restoration that is conventionally cemented or adhesively bonded to the tooth without involving the covering of the cusps in its design. Inlays are often utilised to restore medium to large dental cavities with well-preserved buccal and lingual walls [82].
  In modern dentistry, ceramic inlay restorations are favoured for their longevity, tooth colour fidelity, and morphological stability [83]. Ceramic inlays are most effective for teeth with low occlusal stress since they provide predictable long-term performance [84]. Ceramic inlays may be customised using conventional methods, but digital procedures provide benefits including fewer lab steps and less time-consuming adjustments [85].

Onlays, endocrowns, and partial-coverage crowns may provide cuspal coverage without reducing axial tooth surfaces or subgingival margins. In the literature, these restorations do not seem well categorised, and their purpose and meaning are disputed.

- Research refers to onlays as partial ceramic crowns, occlusal onlays, bonded onlays, and overlays [86,87,88]. These minimally invasive restorations were displayed to be effective for treating major posterior dental defects [82,89]. In order to maintain the amount of remaining healthy tooth structure, bonded onlays are mostly recommended when adequate protection of the tooth cusps is required without the demand for a conventional crown [90]. The preservation of tooth structure is prioritised while achieving both functional and aesthetic goals through the use of onlays, which also serve to reinforce the remaining tooth tissue. Additionally, they may cure extensive occlusal wear and strengthen weakened teeth with fractured or cracked tooth syndrome [91,92]. Bonded onlay cases are prepared with defect-specific, non-retentive, smooth edges and adequate thickness of ceramic materials, unlike conventional MOD onlays [93]. Advantages of onlays include the preservation of natural tooth structure, the reinforcement of weakening cusps, and the delivery of exceptional aesthetics. Contraindications extend to extensive dental decay or structural damage leading to full-coverage restorations and insufficient remaining tooth structure for appropriate retention and stability. It is often bonded using conventional cementing or resin cements. Onlays should be considered when the distance between the buccal and lingual cusp edges is more than the isthmus width or when a cusp indicates weakness [94].
- An overlay is an adhesive-bonded onlay that covers all cusps of a posterior tooth [95]. Ferraris suggests using onlays and overlays for filling cavities, modifying occlusal surface shape, and preserving pulp vitality in cracked teeth [78,94]. The most suitable restoration design, material, and thickness depend on the tooth’s location on the dental arch and the amount of dental material that was previously lost [95]. An adhesive post is optional but not contraindicated for conservative root canal treatment. Different preparation methods are available: butt joint (strict horizontal restriction), bevelled, and overlay (shoulder preparation) [78]. If the cusp is vulnerable, the butt joint, the most typical adhesive procedure, protects it. The bevel, a different butt joint design, increases enough space or enamel surface at the preparation margins for aesthetics. The shoulder is beneficial for cervical grasps but typically recommended for cusp fracture restoration [95]. Overlays can ensure a physiological occlusion is safer and stable. While more pricey than other restoration treatments, they may provide superior control over shape and aesthetics over time. The term “occlusal onlays” implies the vertical dimension of occlusion rising, redefining occlusal relations [87]. Ceramic overlay restorations provide a durable dental treatment with a survival rate above 90%, equivalent to full-coverage crowns. While the most prevalent failure pattern is ceramic and/or tooth fracture (76.2%), it is still a minor proportion compared to the total success rate; these restorations are a durable, safe, and successful option for restoring posterior teeth [96]. The literature describes two overlay preparation designs. The first method involves a complete coverage, non-retentive preparation guided by occlusal anatomy. It also gives sufficient interocclusal clearance for the restorative material. A no-preparation (tabletop) or minimally invasive design is indicated for teeth with significant occlusal wear-induced tissue loss [82,97]. Etienne et al. [95] developed a new categorisation system for overlays, based on remaining tissue support and future restorative material thickness. Type I, most often known as “tabletop”, has a 1.5 mm layer bonded to enamel. In most cases, type II is bonded onto dentin and varies from 1.5 to 4 mm in thickness. Type III is similar to type II but has a restored core with a dentin replacement. Type IV is suited to endodontically treated teeth. Each overlay type has a distinctive therapeutic function and requires various materials and bonding processes [95]. Surface tabletop preparation involves removing merely the incisal or occlusal surface of the tooth structure. This treatment is recommended for incisal edge chipping, mild enamel damage, cosmetic augmentation, minor malalignment and tooth shape discrepancies. Tabletop restorations provide characteristics such as minimal dental structure loss, natural tooth integrity protection, and aesthetic and functional enhancements. This prosthetic preparation is not suggested for severe tooth decay or structural damage that requires extensive tooth preparation and inadequate tooth structure for retention and stability [98].
- Veneerlays or vonlays combine onlay and buccal veneer procedures for dental crown rehabilitation. Veneerlays are often recommended for damaged posterior teeth with both buccal and occlusal surfaces, as well as for patients with occlusal wear [82]. McLaren et al. found that veneers provide advantages over complete crowns, such as less invasiveness, procedure sensitivity, and more repairability [99].
- Porcelain laminate veneers are increasingly a widely used technique able to satisfy particularly high expectations. Furthermore, their therapeutic indications have progressively broadened as a result of the development of novel ceramic materials [100,101]. Veneer preparation requires the use of a custom-made ceramic veneer after a thin layer of enamel is removed from the tooth’s facial surface. This approach is highly recommended for cases of discoloured or stained teeth, cracked or chipped enamel, minor dental misalignment or size or shape anomalies, anterior teeth cosmetic and aesthetic enhancement, and little tooth structure removal that results in significant aesthetic benefits [102]. Veneers have numerous advantages, like long-term aesthetic impacts, minimal tooth structure reduction, and excellent final results. Patients with significant malocclusion that requires orthodontic treatment, inadequate enamel thickness for bonding procedures, or severe tooth decay or structural damage that must undergo full coverage restorations are not encouraged to opt for veneers [103].
- Endocrowns are bonded monolithic ceramic restorations that are indicated for molars subject to endodontic therapy due to significant tissue damage [104]. Additional initial procedures that predominantly depend on biomechanics have to be performed in order to satisfy the requirements. The pulp chamber preparation does not extend into the root canals, and the cervical edge is designed like a butt joint [105]. The approach of bio-integrated prosthesis is consistent with this effective and fundamental idea. Both computer-assisted machining and ceramic material pressing may be used to prepare the restoration [106]. The introduction of this restorative procedure as an alternative to conventional root-anchored restorations is made achievable by new-generation ceramics and adhesives. A particularly biomechanically advantageous restoration process is made possible by the unique preparation method and adhesive bonding [107].

Given the variety of restorative designs and material options currently available, Table 2 provides a concise overview of minimally invasive ceramic restorations, ceramic material selection, and recommended cementation approaches.

### 4.2. Biomechanical and Aesthetic Considerations

Biomechanics focuses on the function and durability of minimally invasive prosthodontic restorations. These restorations are designed to withstand stresses from normal oral activity without damaging the tooth structure. Occlusal stresses, material selection, and design are main factors in achieving biomechanical qualities. High-strength ceramics and hybrid ceramics are ideal for minimally invasive restorations due to their high fracture resistance, strong flexural strength, and wear properties, which mirror natural tooth structure.

The biomechanical performance of minimally invasive restorations is also influenced by their design. Occlusal forces must be uniformly distributed over the whole tooth structure while minimising regions of stress concentration. Conservative dental preparations that preserve the original tooth structure raise the overall biomechanical stability of the restoration [108].

Aesthetics include both the exterior design of restorations and the integration of the surrounding soft tissues. Establishing harmony between the restoration and the adjacent gums is a main determinant for achieving a natural appearance. The emerging profile of the restoration from the gingiva must be carefully analysed and created to replicate the natural contours of the natural teeth [109]. Beyond colour difference, research has increasingly emphasised the importance of translucency-related metrics when evaluating the aesthetic performance of zirconia restorations. The translucency parameter (TP), defined as the colour difference in a material measured against black and white backgrounds, is closely related to human visual perception and represents a clinically relevant indicator of optical behaviour. Recent evidence suggests that TP is strongly influenced by zirconia microstructure, particularly cubic phase content and grain size distribution, which affect light transmission through the material. In addition, the contrast ratio (CR) has been proposed as a complementary optical parameter reflecting the masking potential and opacity of restorative materials. Therefore, this combined assessment may provide a truthful understanding of the aesthetic performance and masking ability of contemporary monolithic zirconia restorations. Furthermore, the development of highly translucent and extra-high-translucent zirconia materials has expanded the indications for monolithic restorations in the anterior area, offering improved optical integration while supporting minimally invasive restorative approaches [110].

From a clinical perspective, the interpretation of colour differences should extend beyond numerical measurements alone. Perceptibility and acceptability thresholds have been proposed as useful reference points for determining whether colour discrepancies are visually detectable and clinically acceptable. Consequently, the aesthetic success of minimally invasive ceramic restorations depends not only on achieving favourable optical properties but also on maintaining colour differences within clinically acceptable limits, thereby facilitating harmonious integration with the adjacent dentition.

#### Preparation Guidelines for Indirect Ceramic Restorations

Successful indirect posterior adhesive restorations demand rigorous control over preparation requirements. To ensure a precise fit, strength, and long-term performance of the treatment, these parameters and considerations must be considered.

- Smooth and rounded ridges and angles:

To minimise ceramic stress, round any sharp points and ridges. This relates to the area where the occlusal isthmus connects to the proximal box and the transition between the lateral walls and the floor [111].

- The minimum thickness:

Current standards specify minimal thickness for different restoration types. Partial and single crowns need 1.5 mm ceramic on the occlusal surface and 1 mm on adjacent margins. Inlays and onlays that have reduced preparations need a minimum thickness of 1 mm for lithium disilicate and 1.5–2 mm for other glass ceramics. A minimum thickness of 0.3 mm is recommended for occlusal surface supplements with preparation (“Table Tops”); however, caution is advised owing to limited clinical experience [111].

- Preparation shapes:

Vertical wall occlusal divergence should start at 6–10°. Ensure clean, linear preparations along the tooth surface to prevent brittle ceramic edges [96].

Furthermore, the surface roughness and brightness of the restorations contribute to their visual uniqueness. Contemporary ceramic processes and materials may provide restorations with surface characteristics that are similar to natural tooth enamel. Minimally invasive restorations need greater technical accuracy compared to standard restorations, demanding competent practitioners and precise attention throughout tooth preparation and bonding procedures [112]. The possible adverse effects of deficient protocols or poor knowledge and skills of a dentist may lead to compromised aesthetics, reduced longevity, or even therapeutic failure.

### 4.3. Longevity, Degradation and Failure Mechanisms

Clinical conditions, material-related issues, and patient-related variables all affect durability. Oral hygiene, parafunctional actions, diet, and lifestyle are all patient-related factors. Material-related aspects include material strength, wear resistance, and bonding properties [112]. Clinical factors consist of tooth preparation, bonding processes, occlusal difficulties, and cementation strategies.

The inadequate comprehension of the chemical, physical, and biological properties of luting materials frequently employed in clinical settings may negatively impact the effectiveness of dental treatments, thereby diminishing the longevity of restorations [113]. The amount of microleakage between the indirect restoration and the tooth structure over time after the restoration is cemented is one of the parameters that determines the clinical consequence of restorative treatment. Increased microleakage is associated with secondary caries, resulting in postoperative sensitivity, weakened pulp integrity, and lowered tooth vitality. Along with dental plaque accumulation, it is important to dedicate every effort to reducing this adverse event [114].

Failures in minimally invasive all-ceramic restorations may occur; nevertheless, meticulous evaluation of the previously described criteria guides optimal outcomes. Fractures or chipping [115], defects in marginal adaptation, bonding failures, secondary caries, wear or erosion of the ceramic material or even incomplete polymerization [116], and aesthetic failures vary among causes of failure in minimally invasive all-ceramic restorations [117]. 

Dentin may be pre-bonded using a dentin-bonding agent immediately after it is cut (immediate dental seal) to minimise the deterioration of bonded surfaces [118]. The permeability of recently formed dentin can potentially be reduced by using a dentin adhesive [119]. A hydrophobic resin coat can help increase the bond strength of simplified adhesives (total-etch, self-etch) by supplementing the hydrophobicity of the adhesive layer. This procedure leaves the adhesive contact area more impermeable to water and reduces the risk of water deterioration [120].

Clinicians may detect and treat these adhesive failure reasons to improve the long-term performance and durability of minimally invasive all-ceramic restorations while reducing the risk of bond failure.

## 5. Conclusions

Adhesive dentistry, advanced ceramic materials, and CAD/CAM technology have reshaped prosthodontics through minimally invasive all-ceramic restorations. The transition from macro-retentive to adhesive-based techniques has enabled considerable preservation of dental tissues without compromising function or aesthetics. 

Long-term success of these treatments is primarily determined by the stability of the adhesive interface, which remains susceptible to hydrolytic processes, procedure sensitivity, and material-dependent conditions. To provide predictable results, clinical protocols—including tooth preparation, surface conditioning, and cementation—must be meticulously managed. 

Ultra-conservative restorative designs, including partial-coverage restorations and occlusal veneers, are clinically achievable due to advances in adhesive systems and restorative biomaterials and bonding performances. However, a clinician’s adherence to evidence-based procedures and case selection are strongly dependent on long-term clinical success.

Future research should focus on optimising the balance between mechanical performance, optical integration, and dental tissue preservation in minimally invasive ceramic restorations. Particular attention should be directed toward the influence of restoration thickness, substrate characteristics, material microstructure, and ageing procedures on long-term clinical performance. Furthermore, prospective clinical studies are needed to validate current laboratory findings and to better define the relationship between adhesive protocols, restorative design, and restoration longevity under functional intraoral conditions.

The relevance of minimally invasive prosthetic techniques in modern restorative dentistry is expected to further enhance as improvements in adhesive systems and ceramic technologies strengthen bond longevity and extend clinical indications.

## Figures and Tables

**Table 1:** Adhesion Strategies for Contemporary All-Ceramic Restorative Materials.

Material Type	Surface Treatment	Adhesion Mechanism	Clinical Considerations
Glass ceramics (feldspathic, leucite, lithium disilicate)	5–10% HF etching + silanisation	Micromechanical retention + chemical bonding via siloxane formation	Requires controlled etching time and proper isolation
Polymer-infiltrated ceramics	Mild HF etching + silane application	Selective matrix dissolution exposing hybrid network	Improves resin infiltration and bonding surface
Zirconia-based ceramics	Air abrasion + MDP primer (APC concept)	Chemical bonding via phosphate monomers (MDP)	No silica → no HF etching effectiveness
Nanoceramic composites	Air abrasion (Al_2_O_3_) + silane/adhesive resin	Micromechanical roughening + hybrid resin infiltration	Dependent on resin matrix content and filler distribution

**Table 2:** Overview of minimally invasive ceramic restorations, material selection, and cementation strategies.

Restoration Type	Indicated Ceramic Materials	Cementation Approach
Veneers	Feldspathic ceramic, leucite-reinforced ceramic, lithium disilicate	Adhesive resin cementation
Inlays	Feldspathic ceramic, leucite-reinforced ceramic, lithium disilicate, hybrid ceramics (polymer-infiltrated ceramic networks and resin nanoceramics)	Adhesive resin cementation
Onlays	Lithium disilicate, zirconia-reinforced lithium silicate (ZLS), zirconia (selected cases), hybrid ceramics (polymer-infiltrated ceramic networks and resin nanoceramics)	Adhesive resin cementation
Overlays, Tabletops	Lithium disilicate, zirconia-reinforced lithium silicate (ZLS), zirconia (selected cases), hybrid ceramics (polymer-infiltrated ceramic networks and resin nanoceramics)	Adhesive resin cementation
Veneerslays, Vonlays	Lithium disilicate, feldspathic ceramic	Adhesive resin cementation
Endocrowns	Lithium disilicate, zirconia-reinforced lithium silicate (ZLS), zirconia, hybrid ceramics (polymer-infiltrated ceramic networks and resin nanoceramics)	Adhesive resin cementation
Partial coverage crowns	Lithium disilicate, zirconia-reinforced lithium silicate (ZLS), zirconia, hybrid ceramics (selected cases)	Adhesive or conventional cementation depending on material and preparation design

## Data Availability

No new data were created or analyzed in this study. Data sharing is not applicable to this article.
